# ‘Spirituality’ and ‘cultural adaptation’ in a Latino mutual aid group for substance misuse and mental health

**DOI:** 10.1192/pb.bp.114.048322

**Published:** 2015-08

**Authors:** Brian T. Anderson, Angela Garcia

**Affiliations:** 1 University of California, San Francisco, USA; 2 Stanford University, Stanford, USA

## Abstract

A previously unknown Spanish-language mutual aid resource for substance use and mental health concerns is available in Latino communities across the USA and much of Latin America. This kind of ‘4th and 5th step’ group is a ‘culturally adapted’ version of the 12-step programme and provides empirical grounds on which to re-theorise the importance of spirituality and culture in mutual aid recovery groups. This article presents ethnographic data on this organisation.

Group Higher Power (a pseudonym, to protect confidentiality) is a mutual aid group in Northern California for Latinos with substance use problems and other mental health concerns. (By ‘Latinos’ we are referring to a very diverse population of persons from Latin America or their descendants. We do not mean in any way to present Latinos as a homogenous group.) It is a ‘4th and 5th step’ group (grupo de Cuarto y Quinto Paso, henceforth ‘CQ’), a variation of the 12-step recovery programme that was founded in Mexico in 1991.^1^ New members join Group Higher Power by completing seven preparation meetings followed by a 2-day *experiencia espiritual* (spiritual experience) in a secluded mountain forest preserve a couple hours from where the group holds its nightly meetings. During the experience, the new members complete the 4th and 5th steps of the 12-step programme: step 4, ‘We made a searching and fearless moral inventory of ourselves’; step 5, ‘We admitted to God, to ourselves, and to another human being the exact nature of our wrongs’.^2^ Afterwards, they are encouraged to attend the nightly meetings, which usually open with the meeting coordinator reading the bye laws of the group, including: ‘Here is a 4th and 5th step group. We are a group of men and women that maintains itself by our own contributions’. In a traditional Alcoholics Anonymous (AA) group, the coordinator would next say: ‘The only requirement for being here is that you wish to stop drinking’. But at Group Higher Power, the coordinator says: ‘The only requirement for being here is that you wish to transcend the pain in your life, and to stop suffering’.

Unlike in AA, CQ members may identify as an *alcohólico* (alcoholic), a *drogadicto* (drug addict), an *enfermo emocional* (emotionally ill, used interchangeably with *neurótico* (neurotic)) or any combination of these. ‘To be an *enfermo emocional*’ is an idiom of distress^3^ that signifies a ‘spiritual’ ailment thought to be at the root of addiction, depression and other ‘neuroses’. The idiom also indicates a correct form of treatment which members learn to engage in and provide to others in the spiritual experience – a dramatic, cathartic style of testimony.

This form of ‘spiritual healing’ can be found in CQ groups like Higher Power located throughout Mexico, the USA, Central America and in some parts of South America and Europe. Because there is no central organisation that unites all CQ groups, it is impossible to know exactly how many there are (see directory of US groups affiliated with the Amor y Servicio branch of CQ: www.amoryserviciousa.org/directorio). That said, our preliminary ethnographic findings suggest that CQ is growing rapidly in Mexico and the USA, making it a community-based mental health resource for potentially thousands of Latinos across North America.

The apparently rapid growth of CQ is important from a public health perspective and interesting for theorising what makes psychotherapy ‘cultural adapted’. Mutual aid groups for addiction are known to be cost-effective interventions.^4^ Latinos in the USA have a disproportionate burden of substance use,^5,6^ and yet they are underserved in the areas of substance use and mental health treatment.^7,8^ There is therefore a pronounced need for accessible, community-based and culturally adapted care for Latinos in the USA.^9,10^ Alcoholics Anonymous may meet this need for some, but whereas Latinos in the USA are thought to recommend AA equally as often as other ethnicities,^11^ Project MATCH^12^ suggests that AA has trouble attracting and retaining Latinos in the USA. Interestingly, Project MATCH also found that, relative to White Americans, Latinos who do attend AA display higher levels of involvement, including ‘God consciousness’,^13^ after out-patient alcohol treatment and several authors have suggested that the incorporation of spiritual and religious elements into culturally appropriate substance use treatment for Latinos merits further examination.^7,12–14^ However, the majority of Latinos in the Project MATCH study sample were south-western Hispanos and it is likely that their responses are not representative of other Latino groups in the USA, such as Puerto Ricans, Mexican–Americans and Mexicans.

Highly spiritual and religious behaviour can already be found in Spanish-speaking AA groups in the USA^15^ and especially in Mexico,^2^ where AA has become over the past several decades the most prevalent source of substance use treatment in the country.^16,17^ But even members of these groups sometimes criticise CQ as being overly religious and refer to CQ derogatorily as a ‘religious sect’. So, if CQ is as popular among USA-based Latinos as our qualitative data suggest, we propose this may be due to how CQ incorporates a culturally appropriate form of spirituality into their recovery programme. In this article, we present ethnographic data on the therapeutic practices of Group Higher Power, which largely resemble those of other CQ groups we have observed in the USA and Mexico. In doing so we attempt to offer a qualitative understanding of ‘spirituality’ in CQ and more specifically, how the ‘spiritual experience’ is configured as a treatment for being *enfermo emocional*. Through this we enquire into how this form of mutual aid spirituality may mediate a successful cultural adaptation of the 12-step programme for underserved Latinos in the USA.

## Data collection and study aims

The ethnographic data presented here come from an ongoing anthropological study of drug addiction, violence and treatment modalities for substance-using Latinos in Mexico and the USA. CQ has been a central focus of our ethnographic fieldwork since January 2013. We have observed and documented the therapeutic practices and living conditions of members of ten CQ groups (4 in Mexico) as well as several other 12-step groups that are specifically for Latinos.^15,16^ We have also conducted dozens of formal and informal interviews with 12-step group members, their family members and health professionals in both countries. Many of the 12-step members had participated in more than one kind of 12-step group over their lifetime (e.g. CQ and AA) and so we were able to gather information on CQ from interviewees who were not currently active CQ members. We have observed one spiritual experience hosted by Group Higher Power. In accordance with exploratory qualitative research methods,^18^ research sites and participants were selected by convenience, as dictated by the opportunities and challenges inherent in maintaining relationships with a dynamic and mobile population over several months. Iterative interpretive analysis of research materials (field notes, interview transcripts, photographs and videos) was conducted to better understand the emic categories of illness, health and healing. This study has been approved by the Institutional Review Board of Stanford University. A draft of this article in Spanish was presented to Group Higher Power, and it is being published with the group's blessing.

## Group Higher Power – characteristics

On its surface, Group Higher Power looks like other Spanish-language 12-step groups in the USA.^15,19,20^ Nightly meetings are held from 19.00 to 21.00 in a suburban storefront in a predominantly Latino neighbourhood. The membership consists of men and women ranging in age from their 20s to their 60s. They mostly hail from Mexico, but some are from Central America and many were born in the USA. Several members are undocumented immigrants. Many speak English, but meetings and informal conversations are conducted in Spanish.

### The setting

The main room of the group has large posters with the 12 steps and 12 traditions of AA, which are in Spanish and hang on the far wall above the desk of the meeting *coordinador* (coordinator) and the podium where members stand and share their testimonials. Between the posters of the 12 steps and the 12 traditions hang framed portraits of Bill W and Dr Bob, the co-founders of AA. The other three walls of the room are adorned with one or two images of Jesus Christ and many more colourful framed certificates and plaques that Group Higher Power has received for giving *compartimientos* (‘sharings’ or testimonies) at the anniversary celebrations of other CQ groups in California, Nevada and Utah. By the dates on the certificates, most groups in this part of the country have only been in existence for 3 to 4 years; a few have been around for up to a decade.

**Fig 1:**
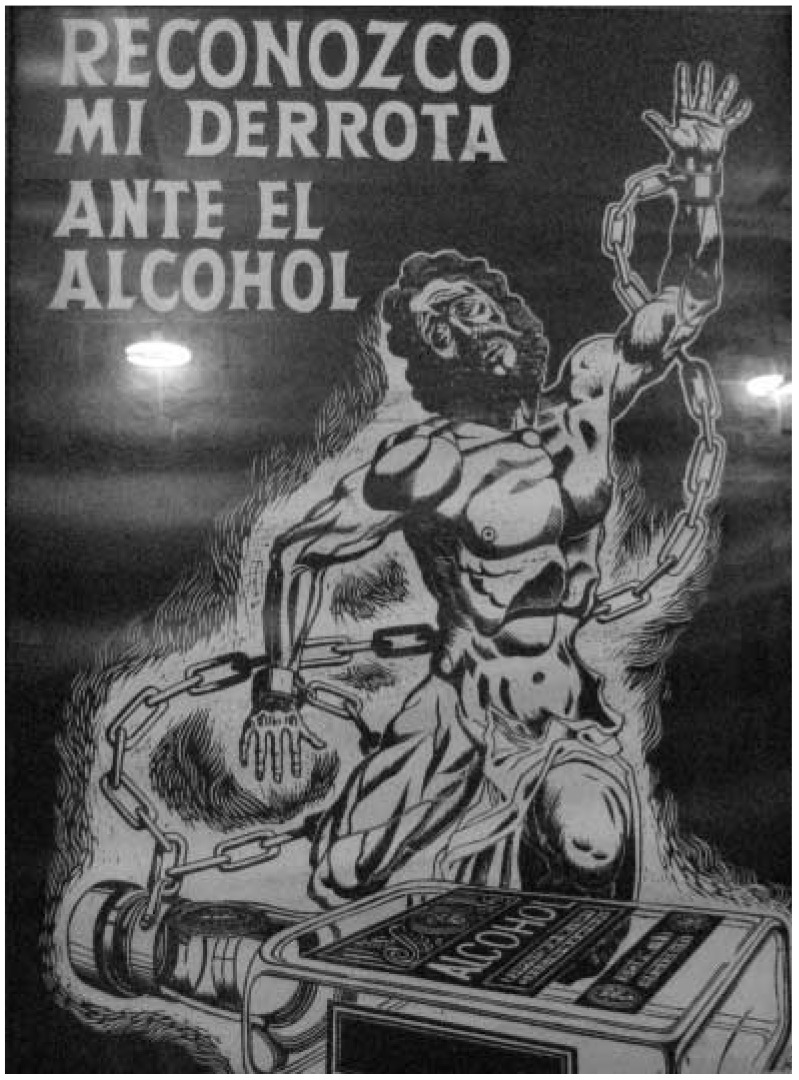
Picture hanging in all 12-step Latino groups' meeting rooms. Photograph by the author

There is a small room in the back where *ahijados* and *ahijadas* (‘sponsees’) can receive *apadrinamiento* (counsel) from their *padrinos* and *madrinas* (sponsors, also known as godfathers and godmothers) in private. And like in so many other Latino 12-step groups, on the wall near the main entrance hangs a black-and-white print of a man, shirtless, dishevelled and shackled, with the words *Reconozco mi derrota ante el alcohol* (I recognise my ruin from alcohol) (Fig. 1).

### Group members

Unlike in Latino AA groups, members of Group Higher Power are explicitly seeking help for more than just their alcoholism; they say they want to cure their ‘emotions’. To be an *enfermo emocional* is to have suffered traumatic and painful events in one's past, often as a child, and then to go through life with the memories of these events smouldering in the unconscious, causing the person to repeatedly hurt others and themselves. This pain is what CQ members say they used to try to avoid when they drank alcohol and used other drugs. The same pain led other members not to use substances, but instead to be ‘neurotic’ or unhappy and self-destructive. Like the ‘dry drunk’ in AA, the *enfermo emocional* displays the same harmful behaviours of an alcoholic who is abstinent but not in recovery, namely lying, being violent and generally suffering from ‘ego’. The prescribed treatment is lifelong dedication to CQ's spiritual path, which starts with completing a spiritual experience.

This is why in the back of the Group Higher Power main meeting room there is another, medium-sized room where the seven preparation meetings are held for newcomers, called *escribientes* (‘writers’), who will soon undergo their first *escritura* (‘writing’, meaning spiritual experience). There is no podium in this room, but there is a wooden desk behind which a coordinator sits, with two other members to his right and left, usually a man and a woman. Some days up to a dozen *escribientes* at a time are being prepared in this room, sitting in their brown, metal folding chairs facing the desk, above which also hang portraits of Bill W and Dr Bob. A couple of *escribientes* typically trickle in late, apologising for not being able to get off of work in time at the factory or cleaning houses. The usual attire is jeans, old tracksuit bottoms, well-worn tennis shoes, but there is also the occasional nice fleece or stylish watch. After everyone is offered coffee or water, the first prayer of the night, the Serenity Prayer, is recited and then the meeting begins.

## The meeting

The members share their testimonies with the *escribientes*, weaving in the benefits they have received from being in the group, like gaining insight into their emotional problems. One man shared: ‘When I came to the group, I had just smoked $100 of crystal meth. I wanted to die … It wasn't until the experience when I realised things were wrong; I had erased those memories. In the experience, I remembered these dark things. I remembered my dad dying, and when I was molested as a child.’

Testimonies also often include mention of a transformation or rebirth in the experience that has helped the person stay sober. It is common for CQ members to admit that they were actually drunk or high when they went for their first experience, but then after that day they no longer needed to drink, smoke or use.

Over the seven preparation meetings, the *escribientes* are gradually introduced to the cathartic style of testimony that is at the very core of CQ's ‘emotional’ therapy. When ‘sharing’ their testimony, members frequently cry, swear and shout, even to the point of sometimes becoming totally distraught. The *escribientes* do not practise giving testimony in the preparation meetings; they do not even speak for the duration of the 2-hour meetings. Instead, they are instructed to sit and reflect on what is being shared with them. Once, after a meeting, an *escribiente* asked the meeting coordinator: ‘Do they always use so many bad words?’ She continued: ‘Because I was thinking about bringing my daughter here, but now I don't know; I don't want her to hear those things’. The coordinator smiled from behind the desk and counselled her with a phrase that is often said at the beginning of the preparatory meetings: ‘If you hear strong language, don't focus on the words, focus on the feelings’. This is the heart of what the *escribientes* are being trained to do – to engage in a reflective, contemplative practice in which they identify with the testimonies of the group members to learn to identify these same emotions in themselves and then release them during the spiritual experience.

### The spiritual experience

At Group Higher Power's spiritual experience each *escribiente* underwent their 4th step by writing a ‘moral inventory’ for about 24-hours straight, with no sleep, little food and lots of coffee. They were instructed to be ‘100% honest’ while answering questions about their lives; these questions are standard across CQ groups and come from the 12-step literature. The 4th step culminated in a celebratory moment in which the *escribientes* and the members held hands in a circle formed around a large wooden cross, crying, reciting prayers and singing Christian hymns in Spanish backed up by a CD playing over a pickup truck's stereo. The *escribientes* were encouraged to have visions of God while they looked up into the starry night sky.

A *padrino* in Mexico City with significant experience in CQ said that the point of the experience is to get the *escribiente* to go through a *colapso a fondo del ego* (‘when the ego hits bottom’) because that is when ‘your spirituality starts to flow’. Group Higher Power members joke that they ‘enter the fourth dimension’ during the experience, meaning that they go into a trance-like state. It is this state that lets them do the emotional healing that they say they cannot achieve with a psychologist, a doctor, not even a traditional AA group. Many cite the spiritual experience as their reason for why they stay in CQ, and why CQ has helped them make behavioural changes that they could not achieve otherwise.

After the experience, the members will attend nightly meetings and continue to rehearse and refine the dramatic testimonial style they learned in the experience. They will stand at the podium, look out of the corners of their eyes, and try to re-enter their painful or shameful memories, recounting them for the group, complete with details of the sights, sounds and smells of what it was like to be there. The meetings are brought to a close with the lights off and everyone standing in a *cadena de amor y servicio* (chain of love and service), holding hands in a large circle. As Christian worship songs are softly played in the background, members are instructed figuratively to leave in the room what they heard and felt that night by whispering it to God. They recite the Serenity Prayer, the AA Responsibility Declaration and the Our Father, and then the meeting is over.

## Discussion

What exactly is it about CQ spirituality that makes this mutual aid organisation especially culturally adapted for Latinos? One observation that seems clear is that the kinds of dramatic public testimony, group prayer and healing and rebirth practices in CQ resemble those of the Pentecostal and Charismatic Catholic movements, which are currently quite popular in Mexico.^21,22^ While true, this does not explain with any real specificity why this would make CQ spirituality congruent with Latinos' cultural frames and therefore a popular choice for those seeking psychological help.

Based on their meta-analysis of psychotherapy studies, Benish *et al*^23^ propose that the key factor for enhancing the cultural adaptability of psychotherapy is the incorporation of an ethnic minority's ‘illness myth’. This suggests that we should consider more closely the CQ idiom of distress, *enfermo emocional*, as a key to what makes CQ culturally adapted for Latinos. CQ emic understandings of being an *enfermo emocional* are sculpted out of a psychodynamic language of neurosis, the unconscious and childhood trauma. The fact that this aetiological discourse is flourishing within a spiritual healing movement might at first seem paradoxical, yet it arguably makes good sense given psychoanalysis' historical ties with religion in Mexico. One of Mexico's better known early champions of Freudian thought, the Catholic monk Gregorio Lemercier, actually attempted in the 1960s to use psychoanalysis to revitalise monastic life,^24^ whose traditions of intensive contemplative practices and spiritual retreats have strong parallels with much of what we see in CQ's modifications and interpretations of the 12-step programme.

The highly ‘spiritual’ nature of being *enfermo emocional* has further implications for CQ's acceptance by Latinos that become even clearer when we consider how 12-step programmes are often criticised for disempowering their members by encouraging them to submit to a higher power and to identify as sick addicts who will forever be in recovery. In CQ, the *enfermo emocional* takes this a step further and is not only eternally in recovery, but they are also intermittently ‘mad’. CQ members sometimes describe their spiritual experience as a form of *locura* (madness); and Mexican AA members and clinicians alike not only allege that CQ's cathartic practices are crude and ineffective, but some have even warned the public against participating in the CQ spiritual experience because of case reports of individuals who have developed psychosis or died by suicide shortly after their experience.

Nevertheless, perhaps it is by making its recovery programme even more ‘spiritual’ than AA that CQ is able to invert these concerns of clinical ineffectiveness and harm, turning the submission to a higher power into a much more positive experience. In Asad's critique of the secular notions of agency and pain,^25^ he delineates how the modern narrative of agency makes clear that agency must be used to avoid suffering (p. 71). Moreover, one who gives into religious ‘emotions’ (glossed ‘passions’) is said to lack the prized agency of a rational subject. To counter these assumptions, Asad pushes us to consider a notion of sanity which, instead of turning on the ideal of self-control, ‘presupposes knowing the world practically and being known practically by it’ (p. 73). He asserts that this ‘allows us to think of moral agency in terms of people's habitual engagement with the world in which they live, so that one kind of moral insanity occurs precisely when the pain they know in this world is suddenly no longer an object of practical knowledge’ (p. 73). According to this alternative understanding of agency, sanity and pain, CQ members could submit to a higher power, enter a state of *locura* in the spiritual experience and dive into the passions of their ‘sick emotions’, and actually thereby maintain, or even regain, their *sano juicio* (‘sanity’), as the 2nd step says can happen (‘[We] Came to believe that a Power greater than ourselves could restore us to sanity’^2^). But for this to be a healthy process requires CQ groups to provide a practical purpose for members to relive their suffering night after night through the testimonies. And hence the prayer circle that ends every nightly meeting and spiritual experience, the *cadena de amor y servicio* (chain of love and service), points up the symbolic importance of service in CQ's practice of spiritual healing. Service, including sharing one's testimony and counselling one's sponsee, is the suture that stitches together CQ sociality and repairs the psychic wounds of the *enfermo emocional*. Given how important sponsorship is in AA in Mexico relative to the USA,^26^ we propose that CQ capitalises on this Mexican proclivity for service to create a mutual aid environment where sectarian notions of agency and suffering can be more fully embodied, thereby allowing CQ members to more adequately respond to the spiritual ‘illness myth’ of the *enfermo emocional*.

Finally, we must stress that reports on the rapid uptake of CQ throughout North America are to this point based on qualitative data alone and they require triangulation with quantitative measures (our research team is currently preparing a survey of CQ groups in Northern California). Moreover, other elements beyond spirituality need to be considered to understand why CQ might be a highly ‘culturally adapted’ form of AA for Latinos. The role of family involvement in CQ should not be underestimated, especially since membership is not restricted to ‘alcoholics’, but can also include ‘drug addicts’ and ‘neurotics’ who do not use substances. Also, larger issues of political economy, state insecurity and violence should not be overshadowed by a narrow interest in ‘cultural adaptability’ when trying to understand why a grassroots treatment modality such as CQ is reported to be growing rapidly in underserved, displaced and marginalised communities. In the neighbourhoods where CQ seems to be growing the quickest, families must deal with poverty, a lack of access to healthcare and the general social fragmentation that Mexico's drug war-related violence has wrought on the country for the past decade. Detailed consideration of these factors is, however, beyond the scope of this article.
